# Lessons from the first-in-human in vivo CRISPR/Cas9 editing of the TTR gene by NTLA-2001 trial in patients with transthyretin amyloidosis with cardiomyopathy

**DOI:** 10.21542/gcsp.2023.4

**Published:** 2023-01-30

**Authors:** Susy Kotit

**Affiliations:** Aswan Heart Centre (AHC), Aswan, Egypt

## Abstract

**Introduction:** Transthyretin amyloidosis (ATTR amyloidosis) is a progressive fatal disease characterized by accumulation of amyloid fibrils composed of misfolded transthyretin (TTR) protein in tissues, resulting in cardiomyopathy and heart failure. Approximately 50,000 people have hereditary ATTR amyloidosis, and up to 500,000 have wild-type ATTR amyloidosis globally, leading to poor quality of life and high morbidity, resulting in death within a median of 2 to 6 years after diagnosis. However, data on the prevalence of ATTR-CM is limited and poorly characterized. NTLA-2001, an in vivo gene-editing therapeutic agent designed to treat ATTR amyloidosis by reducing the concentration of TTR in serum by knocking out the TTR gene, has been shown to be effective, presenting a new therapeutic strategy. However, the safety, tolerability, and pharmacodynamic response to IV NTLA-2001 administration has not been yet demonstrated.

**Study and results:** The first-in-human in vivo CRISPR/Cas9 trial of TTR Gene editing by NTLA-2001 in patients with Transthyretin Amyloidosis and cardiomyopathy was designed to evaluate the safety, tolerability, efficacy, and pharmacokinetic and pharmacodynamic responses to IV NTLA-2001 administration and its effect on serum transthyretin (TTR) levels in patients with ATTR amyloidosis and cardiomyopathy. Twelve subjects received NTLA-2001 (three NYHA I/II subjects at 0.7 mg/kg, three subjects at 1.0 mg/kg, and six NYHA III subjects at 0.7 mg/kg). Serum TTR levels were reduced from the baseline in all subjects (mean>90% after 28 days). Mean % reductions (+/-SEM) from baseline to day 28 were: NYHA I/II at 0.7 mg/kg = 92% (1%), at 1.0 mg/kg = 92% (2%), and for NYHA III at 0.7 mg/kg = 94% (1%) maintained through 4–6 months. Two of the 12 patients (16.7%) reported a transient infusion reaction. One patient experienced a grade 3 infusion-related reaction that resolved without any clinical sequelae.

**Lessons learned:** This study showed a significant and consistent reduction in serum TTR protein levels after a single admission, while being generally well tolerated, representing a potential new option for the treatment and improvement of the prognosis of cardiac ATTR amyloidosis. Further research into the long-term safety and efficacy of NTLA-2001, particularly in higher-risk patients, including continued monitoring of whether knockout of the TTR gene results in sustained TTR reduction over the long term, is essential. Evaluation of the potential effects of markedly reduced TTR levels on patients’ clinical outcomes, with a focus on functional capacity, quality of life, and mortality benefits are essential. The analysis of the use of this technology for an array of other diseases is vital.

## Introduction

Transthyretin amyloidosis (ATTR amyloidosis), is a progressive fatal disease characterized by the accumulation of amyloid fibrils composed of misfolded transthyretin (TTR) protein in tissues, predominantly the nerves and heart^1–4^.

Wild-type (acquired) ATTR amyloidosis (wATTR), the more common type of ATTR is an acquired condition with an unknown underlying cause that results commonly in cardiomyopathy and is an increasingly recognized cause of heart failure^2, 5, 6^. Hereditary ATTR amyloidosis (hATTR), triggered by more than 100 different pathogenic mutations in TTR^7^, has a clinical phenotype dominated by amyloid polyneuropathy or cardiomyopathy, with most patients having a combination of the two^5, 6, 8^.

It is estimated that globally 50,000 persons have hereditary ATTR amyloidosis, and up to 500,000 have wild-type ATTR amyloidosis^9, 10^. Transthyretin amyloid cardiomyopathy (ATTR-CM) leads to poor quality of life, high morbidity, and results in death within a median of 2 to 6 years after diagnosis^11^. In the case of amyloid polyneuropathy in the absence of cardiomyopathy, life-expectancy increases to up to 17 years^12^.

Recent studies suggest that ATTR-CM is responsible for 20% of heart failure (HF) cases presenting with increased myocardial wall thickening ≥14 mm^13, 14^. However, data on the prevalence of ATTR-CM are limited and not well characterized^15^ due to missed and delayed diagnosis^16^ related to the heterogeneous clinical presentation^17–25^ and mainly because of a lack of sensitive diagnostic modality. Advancements in nuclear cardiac imaging with technetium pyrophosphate scan are leading to a more sensitive diagnosis of ATTR-CM, without cardiac biopsy^26^.

The TTR gene is present on chromosome 18. hATTR follows an autosomal dominant inheritance pattern; however, disease penetrance is more complicated and less understood. The age of onset of clinical disease in hATTR-CM varies widely and depends on the type of mutation, which has varied geographical distribution^27^. Wild-type ATTR is possibly related to the aging process as there is an absence of TTR mutation^28^ and the prevalence increases with older age^28, 29^.

Mutations in the *TTR* gene and age-related changes in the structure of the TTR peptide can cause misfolding of the TTR protein, forming insoluble fibers and resulting in amyloid deposits that disrupt normal organ function and lead to progressive organ failure.

In the heart, they occupy interstitial spaces in the myocardium, making it stiff and rigid. TTR deposition causes further myocardial fibrosis and eventually affects its mechanical function. Due to TTR deposition, the myocardium appears thickened and hypertrophied on cardiac imaging.

Compromise in ventricular compliance initially causes diastolic dysfunction. In advanced stages, myocardial dysfunction can result in globally reduced systolic function^30^. Diastolic dysfunction causes an increase in left ventricular end-diastolic pressure and left atrial pressure. Persistently increased left atrial pressure and left atrial dilatation increases the likelihood of developing atrial arrhythmias. Myocardial infiltration also affects the electrical conduction system, resulting in conduction disorders^31^ as ventricular arrhythmias^32^.

**Figure 1.**
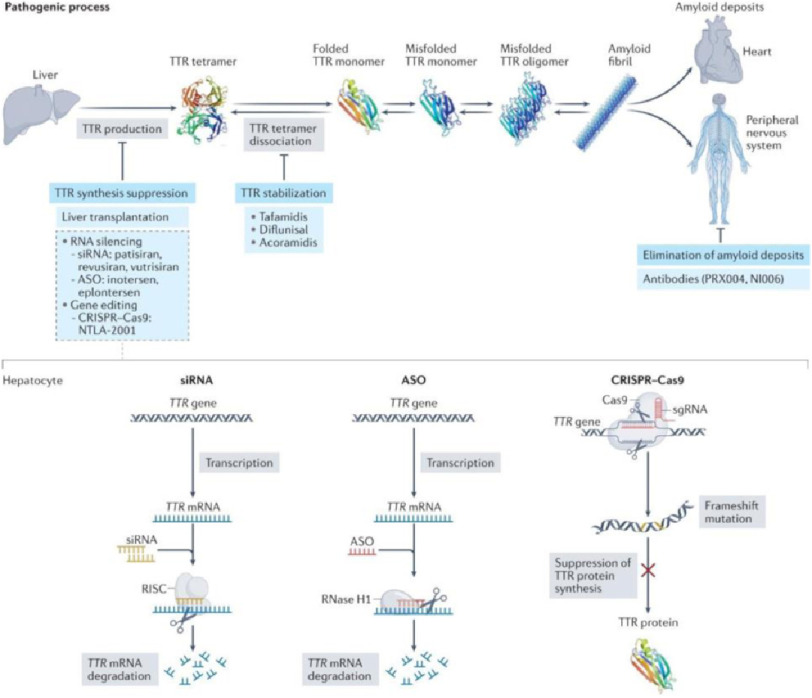
Overview of treatments available for transthyretin amyloidosis and their mechanisms of action. Therapies that aim to inhibit transthyretin production include liver transplantation, small interfering RNA (transthyretin) gene silencers, and DNA editing therapies (CRISPR Cas9). The lower part of the figure summarizes their type of interaction with nucleic acids. The main stabilizing drugs of transthyretin are represented by tafamidis, diflunisal, and acoarmidis. Some antibodies that bind to transthyretin have the potential to trigger immune-mediated amyloidolysis^44^.

The available therapeutic strategies for ATTR, such as diflunisal, and tafamidis, stabilize the TTR protein in its tetrameric form or patisiran and inotersen which inhibit TTR protein synthesis leading to a decrease in the formation of amyloid^33^, postpone progression, relieve symptoms, and prolong survival (Figure 1)^34–36^. However, all therapies require long-term administration to maintain sufficient TTR knockdown, exacerbating adverse effects^37^ while progression is not completely prevented^38^. In addition, due to cost, access to treatment is limited. Despite the availability of TTR protein stabilizers for the treatment of ATTR amyloidosis, significant morbidity and mortality persist^39^. Consequently, there is an unmet need to develop therapies that can halt or reverse disease, even in advanced ATTR, and have an improved route and frequency of administration, given the chronic nature of the disease.

Recently, clinical trials investigating chronic therapy with gene silencing agents targeting mRNA have shown that lowering TTR protein levels results in cardiac benefits. NTLA-2001 is an in vivo gene-editing therapeutic agent designed to treat ATTR amyloidosis by reducing the concentration of TTR in the serum by knocking out the *TTR* gene. This novel CRISPR (clustered regularly interspaced short palindromic repeats) and combined Cas9 (Cas9 endonuclease) system comprises a lipid nanoparticle (LNP) encapsulating mRNA for Cas9 protein and a single guide RNA targeting the TTR gene^40^. The CRISPR/Cas9 gene-editing technology^41^ allows the genome to be altered by causing a double-stranded DNA break^42^.

**Figure 2.**
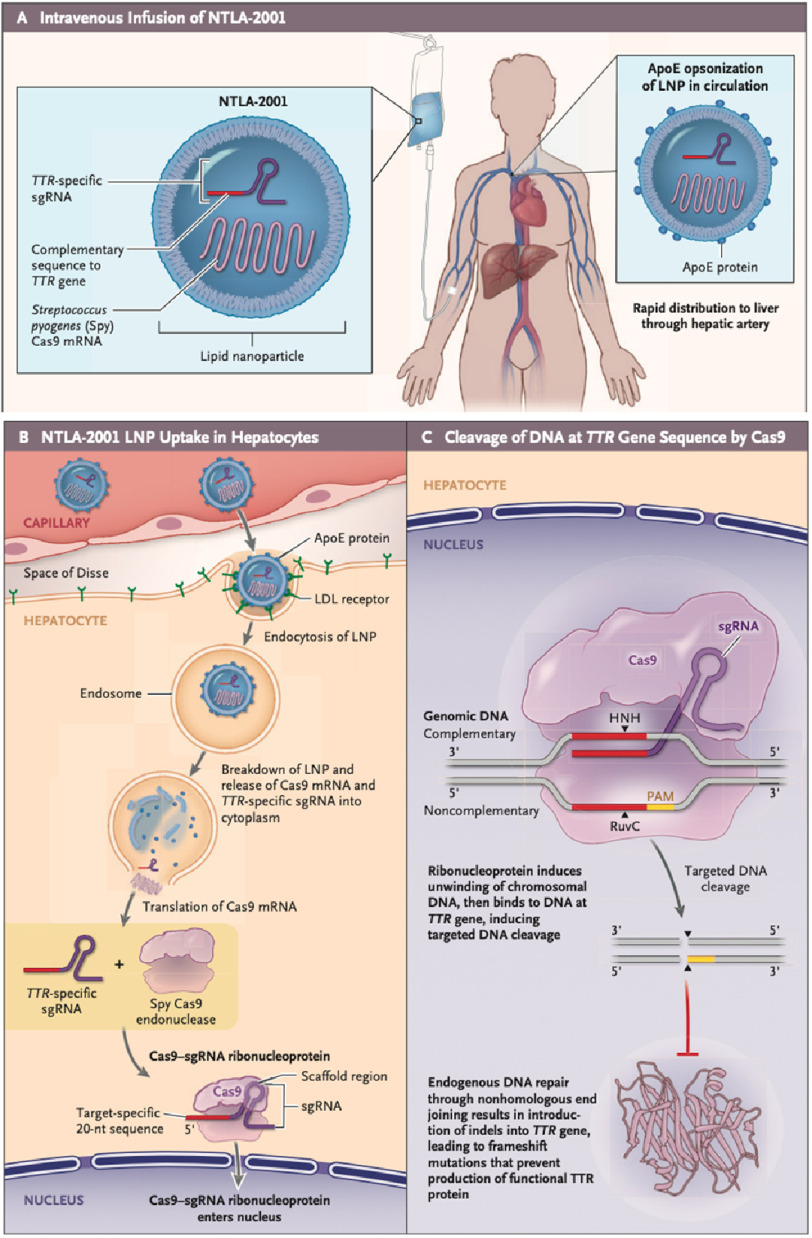
Mechanism of action of NTLA-2001. (A) shows the primary components of NTLA-2001. The carrier system for NTLA-2001 is a lipid nanoparticle (LNP). The LNP is based on a proprietary ionizable lipid, combined with a phospholipid, a PEGylated lipid (molecular weight of polyethylene glycol, 2000 Da), and cholesterol, formulated in an aqueous buffer for intravenous administration. The active components of NTLA-2001 are a human-optimized messenger RNA (mRNA) molecule encoding *Streptococcus pyogenes* (Spy) Cas9 protein (an approximately 4400-nucleotide sequence with a molecular weight of approximately 1.5 MDa) and a single guide RNA (sgRNA) molecule (molecular weight of approximately 35 kDa) specific to the human gene encoding transthyretin (TTR). These components form the cargo of the LNP for drug administration. After intravenous administration of NTLA-2001 and entry into the circulation, the LNP is opsonized by apolipoprotein E (ApoE) and transported through the systemic circulation directly into the liver, where it is preferentially distributed. (B) shows transport of the NTLA-2001 LNP into the capillaries of the hepatic sinusoids inside the liver. As with other clinically approved LNPs,^27^ NTLA-2001 is then expected to undergo uptake by the low-density lipoprotein (LDL) receptor expressed on the surface of the hepatocytes, followed by endocytosis and endosome formation. After breakdown of the LNP and disruption of the endosomal membrane, the active components (the *TTR*-specific sgRNA and the mRNA encoding Cas9) are released into the cytoplasm. The Cas9 mRNA molecule is translated through the native ribosomal process, producing the Cas9 endonuclease enzyme. The *TTR*-specific sgRNA interacts with the Cas9 endonuclease, forming a clustered regularly interspaced short palindromic repeats (CRISPR)–Cas9 ribonucleoprotein complex. (C) shows that the Cas9 ribonucleoprotein complex is targeted for nuclear import and enters the nucleus, where it recognizes the protospacer-adjacent motif (PAM) on the non-complementary DNA strand in *TTR*. A target-specific 20-nucleotide sequence at the 5’ end of the sgRNA binds to the DNA double helix at the target site, allowing the CRISPR-Cas9 complex to unwind the helix and access the target gene. Cas9 undergoes a series of conformational changes and nuclease domain activation (HNH and RuvC domains), resulting in DNA cleavage that is precisely targeted to the *TTR* sequence, as defined by the sgRNA complementary sequence. Endogenous DNA-repair mechanisms ligate the ends of the cut, potentially introducing insertions or deletions of bases (indels). The generation of an indel may result in the reduction of functional target-gene mRNA levels as a result of missense or nonsense mutations decreasing the amount of full-length mRNA, ultimately resulting in decreased levels of the target protein. Indels that result in abrogated production of the target protein, in this case TTR, are termed knockout mutations^40^.

NTLA-2001, administered by a single intravenous infusion, is taken up by LDL receptors on hepatocytes, followed by endocytosis of the particle. The Cas9 mRNA is translated into the cytoplasm. The formed Cas9 protein subsequently interacts with the sgRNA targeting the TTR gene. This complex enters the nucleus, where the sgRNA binds to the complementary sequence of the DNA. Endogenous DNA repair subsequently leads to indels in the TTR gene that prevent the production of the TTR protein. Importantly, knock out of the gene coding the TTR protein minimally affects the normal physiological processes (Figure 2)^43^.

In a small group of patients with hereditary ATTR amyloidosis and polyneuropathy, administration of NTLA-2001 led to decreases in serum TTR protein concentrations through targeted knockout of TTR with only mild adverse events^40^ providing clinical proof of concept for in vivo CRISPR-Cas9–mediated gene editing as a therapeutic strategy. However, the safety, tolerability, and pharmacodynamic response to IV NTLA-2001 administration to knock out the TTR gene and reduce serum TTR in subjects with ATTR amyloidosis with cardiomyopathy has not yet been demonstrated.

## The study

This study presents the first-in-human in vivo CRISPR/Cas9 (phase 1) trial (NCT04601051) of TTR Gene editing by NTLA-2001 (Intellia/Regeneron) in patients with Transthyretin Amyloidosis and cardiomyopathy^45–47^.

The study is part of a two-part, open-label, multi-center Phase I randomized clinical trial designed to evaluate the safety, tolerability, efficacy, and pharmacokinetic and pharmacodynamic response to IV NTLA-2001 administration and its effect on serum transthyretin (TTR) levels in patients with ATTR amyloidosis with cardiomyopathy. The primary endpoints included adverse events, dose-limiting toxicities, and changes from baseline serum TTR levels at seven days to six months post-infusion.

A total of 12 patients (median age, 75 years; all male) were enrolled in the cardiomyopathy arm of the NTLA-2001 phase 1 study. Enrolled patients had either hereditary transthyretin amyloidosis with cardiomyopathy (hATTR-CM) or wild-type cardiomyopathy (wATTR-CM) and NYHA class I to III. All patients received a single dose of NTLA-2001 administered via intravenous infusion.

Two doses (0.7 mg/kg and 1.0 mg/kg) were evaluated in subjects with NYHA Class I/II heart failure and one dose (0.7 mg/kg) in subjects with NYHA Class III heart failure.

Circulating serum TTR was measured in samples collected at baseline, days 7, 14, and 28, and at pre-determined time points thereafter until study completion (2, 4, and 6months).

## Results

Twelve subjects received NTLA-2001 (3 NYHA Class I/II subjects at 0.7 mg/kg and 3 subjects at 1.0 mg/kg, and 6 NYHA Class III subjects at 0.7 mg/kg).

Serum TTR levels rapidly and profoundly reduced from baseline in all subjects (mean >90% at 28 days). Mean % reductions (+/-SEM) from baseline to day 28 were: NYHA Class I/II at 0.7 mg/kg = 92% (1%), at 1.0 mg/kg = 92% (2%), and for NYHA Class III at 0.7 mg/kg = 94% (1%) (Table 1). This reduction was maintained through months 4-6.

**Table 1:** Results of the first human trial of gene editing in vivo (adapted from ^47^).

	**NTLA-2001 (0.7 mg/kg)** NYHA Class I/II HF (*n* = 3)	**NTLA-2001 (1.0 mg/kg)** NYHA Class I/II HF (*n* = 3)	**NTLA-2001 (0.7 mg/kg)** NYHA Class III HF (*n* = 6)
Primary endpoints			
Adverse events, dose-limiting toxicities	None or mild	None or mild	Single Grade 3 infusion-related event that resolved without clinical sequelae
Serum TTR (Mean % reduction +/- SEM)	92% (1%)	92% (2%)	94% (1%)

**Notes.**

Results: First ever human trial of gene editing *in vivo.* Mean TTR reduction >90% across both doses by day 28, sustained 4-6 mo. NTLA-2001 was generally well tolerated with a similar result in NYHA Class I/II or III HF.

Two of the 12 (16.7%) patients reported transient infusion reactions, which were the only observed treatment-related adverse events. One patient with NYHA class III who received a dose of 0.7 mg/kg experienced a grade 3 infusion-related reaction, which resolved without any clinical sequelae. No other patients reported a treatment-related adverse event. No clinically significant laboratory abnormalities were observed at either dose level.

## Discussion

In this first-ever human trial of gene editing in vivo, a single intravenous infusion of NTLA2001, the novel CRISPR/Cas9-based gene editing therapy, significantly reduced circulating transthyretin (TTR) protein levels in patients with ATTR amyloid cardiomyopathy. All patients achieved over 90% reduction in serum TTR levels by day 28, sustained through 4–6 months, regardless of baseline NYHA class, indicating consistent and durable TTR reduction at both 0.7 and 1.0 mg/kg doses of NTLA-2001.

Moreover, NTLA-2001 gene editing therapy was generally well tolerated, with mild and transient infusion reactions being the only adverse events observed, with no clinically significant laboratory findings after infusion.

Reductions in TTR levels over 90% in this trial were achieved with higher doses than those used in the previously reported polyneuropathy arm of the study^40^; however, there were no significant adverse events.

Recently, the APOLLO-B trial of patisiran in patients with ATTR amyloidosis with cardiomyopathy showed an average 87% serum TTR level reduction, but with the need for intravenous infusions every 3 weeks, life-long^48–50^, which is a disadvantage compared to the single-dose admission of NTLA-2001.

One possible concern regarding the use of CRISPR technology in humans is the potential for off-target effects^51^, which has been addressed with a very rigorous process when selecting the guide RNA, targeting the specificity of the TTR gene. Furthermore, various studies using primary human hepatocytes found no evidence of off-target editing^52, 53^ at concentrations of NTLA-2001 that were threefold greater than the concentration used to knock down the protein by 90%^54^, indicating that the specificity of NTLA-2001 for the TTR gene seems to be absolute.

The limitation to the interpretation of the current study is that it presents only the initial data of a Phase 1 dose escalation study in a small cohort of patients from which no dosing guidelines or long-term data can be deduced. Consequentially, the dose-expansion portion of the study (Part 2) which will inform the dose selection for subsequent pivotal studies is to be completed by the end of 2022^45, 46^.

Overall, the significant and consistent reductions in serum TTR protein levels observed in this study indicate that IV NTLA-2001 is a potential new treatment option that may stop disease progression in patients with ATTR amyloid cardiomyopathy and potentially improve prognosis and mortality.

Further research into the role of CRISPR in ATTR therapy is crucial for establishing a permanent cure for this disease. Larger studies are needed to analyze the long-term safety and efficacy of NTLA-2001 admissions, particularly in high-risk patients, such as those in NYHA class III. Continued monitoring of whether knockout of the TTR gene results in sustained long-term TTR reduction is essential. In addition, the potential effects of markedly reduced TTR levels on patients’ clinical outcomes were evaluated, with a focus on functional capacity, quality of life, and mortality benefits.

The data obtained in this study demonstrate promising perspectives for CRISPR and Cas9-based in vivo gene editing in humans. The use of this technology for an array of other diseases should be analyzed, not only to knock out the expression of harmful protein products, but also to insert genes to produce functional proteins where mutations cause pathologic deficiencies.

## Lessons learned

This first-ever human trial with the investigational CRISPR/Cas9-based in vivo gene editing therapy NTLA-2001 showed a significant and consistent reduction in serum TTR protein levels after a single admission and was generally well tolerated, representing a potential new option for the treatment and improvement of prognosis of cardiac ATTR amyloidosis.

Further research into the long-term safety and efficacy of NTLA-2001 is necessary, particularly in higher-risk patients, such as those in NYHA class III. Continued monitoring of whether knockout of the TTR gene results in sustained TTR reduction over the long term is required. Evaluation of the potential effects of markedly reduced TTR levels on patients’ clinical outcomes, with a focus on functional capacity, quality of life, and mortality benefits are essential.

The data provided in this study demonstrates promising perspectives for CRISPR and Cas9-based in vivo gene editing in humans, and should inspire analysis of the use of this technology for an array of other diseases.
